# Gastrointestinal Cancer: Outcomes and Therapeutic Management

**DOI:** 10.3390/jcm14217541

**Published:** 2025-10-24

**Authors:** Evgenia Mela, Maximos Frountzas

**Affiliations:** 1 First Propaedeutic Department of Surgery, Hippocration General Hospital, School of Medicine, National and Kapodistrian University of Athens, 15772 Athens, Greece; vemela@hotmail.com; 2 Department of Colorectal Surgery, The Royal Marsden Hospital NHS Foundation Trust, London SW3 6JJ, UK

## 1. Evolving Frontiers in Gastrointestinal Oncological Care

In recent years, a remarkable transformation has been noticed in the management of gastrointestinal malignancies [1]. Advances in imaging techniques, molecular profiling, and risk stratification models are progressively transforming the treatment of oncological patients towards unprecedentedly personalized approaches [1,2]. Such advances herald a novel era in precision oncology, characterized by developments not only in systemic therapies, which include targeted agents and immune checkpoint inhibitors, but also in surgical and multidisciplinary approaches [1,3]. Minimally invasive techniques, endoscopic procedures, enhanced recovery after surgery (ERAS) protocols, and prophylactic surgery for genetically predisposed populations are incorporated into clinical practice, illustrating the shift where innovation serves both survival and patients’ quality of life [4,5].

In this context, there is growing awareness of factors affecting patient outcomes beyond tumor biology and stage. Diagnostic delays, particularly intensified during healthcare crises and perioperative changes in microcirculation and inflammatory responses, along with socioeconomic factors and healthcare system limitations, are acknowledged as significantly impacting patients’ quality of life and prognosis [6,7,8]. These findings emphasize the importance of tailoring treatment strategies to the individuals and the need for research to focus on minimally invasive and multimodal approaches while it bridges developments in technology with patients’ needs and expectations.

## 2. Insights from This Special Issue

The contributions to this Special Issue, titled “Gastrointestinal Cancer: Outcomes and Therapeutic Management”, provide valuable perspectives on the shifting landscape of gastrointestinal cancer care, reflecting the multifaceted nature of gastrointestinal oncology spanning the continuum from prevention to palliation. Fugǎrețu et al. conducted a systematic review on the role of prophylactic gastrectomy and endoscopic surveillance in patients with proximal polyposis of the stomach (GAPPS) [9]. Considering the lack of consensus on diagnosis and treatment, along with the poor understanding of GAPPS’s natural history, it was emphasized that the decision for prophylactic surgery should be made on an individual basis. However, the diagnosis of dysplasia represents a clear indication for a prophylactic gastrectomy. Similar to inherited cancer syndromes, rare malignancies pose significant diagnostic challenges. Szkudlarek et al. described a rare case of medullary carcinoma of the small intestine diagnosed during the COVID-19 pandemic [10]. Their study demonstrated the diagnostic delays during the pandemic, underlining also the pivotal role of a high level of clinical suspicion and the use of immunohistochemistry methods for the diagnosis of rare gastrointestinal tumors.

While early diagnosis is important in improving prognosis in gastrointestinal oncology, the postoperative course, which is influenced by perioperative biological responses, also has an impact on prognosis. Drinhaus et al. examined perioperative shedding of Syndecan-1 and veno-arterial CO_2_ gap in patients undergoing esophagectomy, revealing that microcirculatory changes transpire despite stable macrocirculatory conditions [11]. Nevertheless, these changes were not predictive of postoperative complications. In addition, Lianos et al. investigated the association of gut microbiota and anastomotic leakage after colorectal resection, revealing reduced alpha diversity and taxonomic differences in patients with anastomotic leak, thereby indicating potential underlying pathophysiologic pathways and the need for future translational studies [12].

Aside from perioperative biological mechanisms, outcomes in gastrointestinal oncology are significantly influenced by population-level factors and demographics. Sawaid et al., in particular, investigated colorectal cancer management in the West Bank, identifying high rates of late-stage diagnoses and mortality along with limited availability of advanced treatments, underlining the urgent need for the establishment of a screening program and the development of treatment guidelines [13]. In addition, Tsokkou et al. examined sex-related differences in colorectal cancer, demonstrating that disparities arise from a combination of genetic, immunologic, and clinical factors [14]. Male patients had a greater incidence and mortality rate from colorectal cancer, more frequently presenting with early left-sided disease, while female patients presented with advanced right-sided tumors with aggressive biological characteristics. These findings suggest the integration of gender-specific factors into prevention, screening, and therapeutic management.

Gingrich et al. shifted attention to palliative interventions in advanced gastric cancer, conducting a systematic review that evaluated the role of palliative gastrectomy, bypass surgery, and endoscopic stenting [15]. Palliative gastrectomy was associated with better survival in selected patients who received adjuvant chemotherapy. On the contrary, endoscopic stenting provided rapid symptom alleviation and initiation of oral intake, shorter hospitalization, and low periprocedural mortality, rendering it an alternative palliative treatment for patients who are not candidates for surgery. These findings underline the significance of individualized decision-making, highlighting the need to align therapeutic decisions with both survival benefit and quality of life. Jang et al. studied palliative chemotherapy for unresectable pancreatic cancer in Korea, finding that older age, male sex, comorbidities, and the omission of radiotherapy or second-line chemotherapy were correlated with greater mortality [16]. Although the FOLFIRINOX regimen yielded better survival outcomes, the lack of staging, performance status, and toxicity requires cautious interpretation and further studies for the assessment of novel second-line treatments, such as nanoliposomal irinotecan and immunotherapy. In the same context of metastatic pancreatic cancer, Rahnea-Nita et al. reported a case of intestinal-type pancreatic adenocarcinoma with a three-year survival rate following the administration of multiple lines of chemotherapy [17]. The report suggests that histological subtype and favorable clinical factors may represent prognostic factors in stage IV pancreatic cancer and require further investigation. Finally, Nakayama et al. examined immune checkpoint inhibitor treatment in patients with advanced esophageal cancer and dysphagia [18]. Their retrospective study on immunotherapy combined with chemotherapy or dual immunotherapy demonstrated higher response rates with improvement in dysphagia. Nevertheless, immune-related adverse events were more frequent than in previous clinical trials, underscoring the need to balance therapeutic benefit against toxicity.

## 3. Emerging Directions of Gastrointestinal Oncology

The evolving landscape of gastrointestinal oncology leads to a future where individualized clinical decision-making represents the standard of care. Advancements in molecular profiling, multiomics data interpretation, and integration are driving surgical oncology beyond the contemporary Halstedian concept and toward a more biologically and clinically informed, risk-stratified framework [2,19,20]. In this context, artificial intelligence is revolutionizing the field by accelerating the aforementioned shift with applications in endoscopic detection, prediction of treatment response, and histopathological diagnosis [21]. Concurrently, the role of the gut microbiome has arisen in gastrointestinal oncology. Differences in taxonomic composition and alterations in microbial diversity have been linked to postoperative complications alongside differentiations in treatment response to systemic treatments [22,23].

Advances in minimally invasive surgery, endoscopic interventions, and ERAS protocols are equally transformative, ushering in a new era of reduced morbidity and shorter length of hospital stay, with a focus on improving patients’ quality of life [4,5,24]. Nevertheless, the adoption of these innovations in surgical and systemic treatments remains complex and unequal, heavily impacted by socioeconomic disparities, and primarily restricted to Western populations [25,26]. However, the development of accessible screening programs, resource-stratified treatment guidelines, and artificial intelligence decision assistance tools could ensure more equal access to gastrointestinal oncological care worldwide [27,28,29].

Therefore, the future of gastrointestinal oncology is being shaped by multiple patient-centered advancements in the realms of microbiome research, artificial intelligence, and minimally invasive surgical techniques. These advances are thus introducing a novel era of gastrointestinal oncology where precision guides innovation, equity is prioritized, and individualized therapeutic decisions constitute the standard of care.
